# Pancreaticoduodenal Arcade Aneurysm Secondary to Dissection of the Superior Mesenteric Artery: A Case Report

**DOI:** 10.3400/avd.cr.25-00063

**Published:** 2025-10-24

**Authors:** Seishiro Akinaga, Takaaki Maruhashi, Yutaro Kurihara, Koyo Suzuki, Yasushi Asari

**Affiliations:** Department of Emergency and Critical Care Medicine, Kitasato University School of Medicine, Sagamihara, Kanagawa, Japan

**Keywords:** pancreaticoduodenal aneurysm, superior mesenteric artery, median arcuate ligament syndrome

## Abstract

Pancreaticoduodenal aneurysms are commonly associated with narrowing of the celiac artery, although involvement of the superior mesenteric artery (SMA) is rare. A 77-year-old man presented with a 5-day history of abdominal and back pain. Contrast-enhanced computed tomography revealed hemorrhagic ascites, a pancreatic arcade aneurysm, and dissection of the SMA. Endovascular embolization was performed using metallic coils. Follow-up imaging demonstrated isolation of the aneurysm and improvement in the shrunken true lumen of the dissected SMA. This case suggests that narrowing of the SMA may alter blood flow in the pancreatic arcade and contribute to aneurysm formation.

## Introduction

Pancreaticoduodenal aneurysms are rare, accounting for approximately 2% of all visceral aneurysms,^1,2)^ and rupture of these aneurysms can be fatal.^2,3)^ Most pancreaticoduodenal aneurysms are true aneurysms. Compared with other visceral artery aneurysms, however, they have a higher risk of rupture, even when their diameter is small. Therefore, treatment is recommended once a diagnosis is established. Endovascular treatment has been shown to be effective in both preventing rupture and managing ruptured aneurysms.^2,4)^ Many reports have indicated that 50%–80% of pancreaticoduodenal artery aneurysms are associated with celiac artery (CA) stenosis.^5)^ However, there are very few reports of pancreaticoduodenal artery aneurysms associated with superior mesenteric artery (SMA) stenosis. In this report, we describe a case of a ruptured pancreaticoduodenal artery aneurysm associated with stenosis at its origin due to dissection of the SMA.

## Case Report

A 77-year-old man receiving treatment for hypertension visited a local physician with a chief complaint of abdominal and back pain that had persisted for 5 days. Contrast-enhanced computed tomography (CT) suggested intra-abdominal bleeding, and the patient was referred to our hospital for further evaluation and management. Upon arrival, his abdominal and back pain had subsided. He was alert and conscious, with a respiratory rate of 16 breaths/min, SpO_2_ of 100% on room air, heart rate of 112 bpm, and blood pressure of 208/97 mmHg. The abdomen was flat and soft, with no rebound tenderness or muscular guarding. Laboratory tests showed leukocytosis, with a white blood cell count of 17100/μL and a mildly elevated creatinine level of 1.19 mg/dL. Hemoglobin was 14.2 g/dL, and no anemia was noted.

**Fig. 1:**
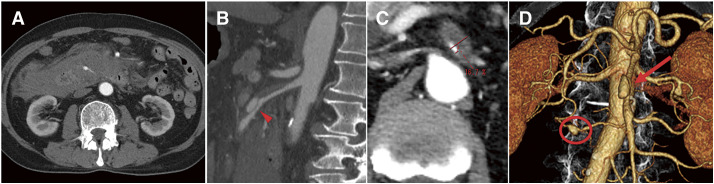
Contrast-enhanced computed tomography on initial presentation. (**A**) Axial view: Hemorrhagic ascites is noted around the pancreas. (**B**) Sagittal view (arterial phase): A false lumen-type dissection is observed at the origin of the superior mesenteric artery, measuring 5.5 cm in length, with narrowing of the true lumen (arrowhead). (**C**) Axial view of the SMA stenosis: The degree of SMA stenosis was calculated to be 83.3%. The percentages indicate the ratio of the true lumen to the overall vascular diameter. (**D**) Three-dimensional reconstruction of the abdominal artery: Dissection of the superior mesenteric artery is visualized (arrow). An aneurysm measuring 12.1 × 8.4 mm is identified in the anterior superior pancreaticoduodenal artery (circle), considered the source of bleeding. SMA: superior mesenteric artery

Review of contrast-enhanced CT scans from the referring hospital revealed a large volume of hemorrhagic ascites and an aneurysm in the pancreatic arcade. The ascites was located around the descending and horizontal portions of the duodenum (**Fig. 1A**), and an aneurysm was identified within the hematoma without evidence of active contrast extravasation. No hematoma was observed around the site of the SMA dissection. Additionally, a dissection was identified at the origin of the SMA, with partial fusiform dilatation in the distal segment. The true lumen of the SMA appeared narrowed at its origin. The stenosis rate at the most narrowed part of the SMA was estimated to be approximately 83%. However, there were no obvious areas of poor imaging in the intestine, and it was determined that intestinal ischemia had not occurred due to blood flow from the true lumen and the CA. The false lumen of the SMA was patent and the surrounding fat density increased, and no vascular remodeling pattern was observed (**Figs. 1B** and **1C**). A diagnosis of ruptured pancreatic arcade artery aneurysm due to acute SMA dissection was made, and endovascular treatment was selected.

**Fig. 2:**
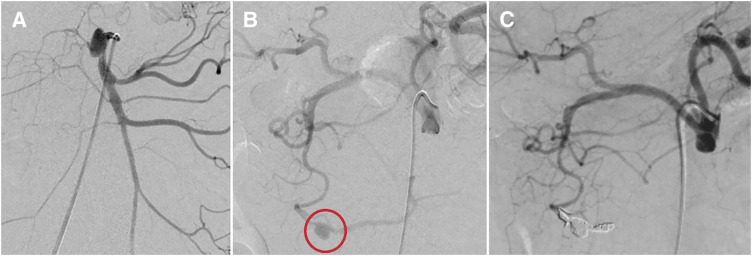
Angiographic findings and endovascular treatment. (**A**) SMA angiography: Dissection is noted at the origin, with the dissection cavity appearing aneurysmal, consistent with computed tomography findings. (**B**) CA angiography: An aneurysm (circle) is visualized in the anterior superior pancreaticoduodenal artery. No contrast extravasation or active bleeding is observed. (**C**) Endovascular treatment: A 5-Fr long sheath was inserted, and a Shepherd’s hook catheter was placed in the CA. Using a microcatheter, the aneurysm was isolated distally with metallic coils. CA: celiac artery; SMA: superior mesenteric artery

CA angiography showed a 12.1 × 8.4 mm aneurysm in the anterior superior pancreaticoduodenal artery without contrast extravasation. SMA angiography revealed dissection-related stenosis at the origin, with post-stenotic dilation. No other vascular abnormalities or stenoses were detected, and segmental arterial mediolysis was ruled out (**Figs. 2A** and **2B**). The aneurysm was approached via the CA and embolized using a metal coil (Target XL; Stryker, Kalamazoo, MI, USA) with a coaxial technique, isolating the aneurysm from the distal side (**Fig. 2C**).

**Fig. 3:**
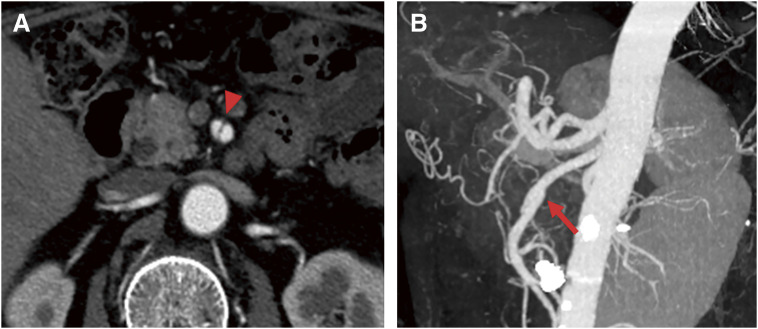
Follow-up computed tomography 2 years after endovascular treatment. (**A**) Axial view at the pancreatic level: The dissection of the superior mesenteric artery persists (arrowhead). (**B**) 3D reconstruction of the abdominal vasculature: The false lumen remained partially patent (arrow) but had shrunk significantly compared with **Fig. 1B**, and no additional intervention was required.

Follow-up contrast-enhanced CT performed on hospital day 5 showed resolution of the intra-abdominal hematoma and disappearance of the aneurysm. Although the dissection of the SMA persisted, there was no expansion of the dissected segment, and the true lumen diameter had improved. The change in imaging findings since the initial visit suggested that the SMA dissection was of acute onset. Therefore, conservative follow-up without further intervention was chosen. The patient’s postoperative course was uneventful, and he was discharged on hospital day 7 (**Fig. 3**). Vasculitis markers, including antineutrophil cytoplasmic antibody, were negative, and no other vascular lesions were identified, leading to a diagnosis of isolated SMA dissection.

## Discussion

Stenosis or occlusion of the CA has been reported as a cause of pancreaticoduodenal aneurysm formation.^5)^ The main contributing factors include atherosclerotic arterial stenosis or compression from median arcuate ligament syndrome.^6)^ Other potential causes of stenosis include trauma, vasculitis, and segmental arterial mediolysis. Stenosis of the CA leads to a compensatory increase in blood flow through the pancreaticoduodenal arcade. The resulting formation of collateral circulation and increased blood flow weakens the arterial wall, potentially causing aneurysms, as reported in previous studies.^7)^

In this case, there was no evidence of CA stenosis or occlusion; however, stenosis at the origin of the SMA due to dissection was observed. Angiographic findings showed delayed contrast enhancement of the SMA and retrograde filling through the pancreatic arcade, indicating significant hemodynamic blood flow restriction. These findings suggest decreased antegrade blood flow in the SMA. It was considered that the narrowing at the SMA origin altered hemodynamics in the pancreatic arcade, contributing to aneurysm formation. A few reports have described pancreaticoduodenal aneurysms associated with SMA stenosis or occlusion.^8)^ Furthermore, there is a past report of pancreaticoduodenal artery aneurysm rupture occurring shortly after hemodynamic changes, such as occlusion of the CA.^9)^ These findings suggest that not only CA stenosis or occlusion but also SMA stenosis or occlusion may increase blood flow through the pancreaticoduodenal arcade and contribute to aneurysm formation.

There is currently no consensus on the need for revascularization of the CA or SMA. In cases of pancreaticoduodenal artery aneurysms associated with median arcuate ligament syndrome, surgical intervention may be indicated due to symptoms such as abdominal pain caused by ligament compression. However, revascularization was not deemed necessary in the present case. Previous reports have shown that safe treatment is possible without CA revascularization and without risk of rebleeding.^10)^ However, no reports exist on secondary preventive strategies for pancreatic arcade aneurysms caused by SMA stenosis or occlusion. In this case, conservative treatment was selected due to improvement in SMA stenosis secondary to dissection. No recurrence was observed during approximately 2 years of follow-up. However, strategies for secondary prevention of pancreatic arcade aneurysms related to SMA stenosis remain to be established. Further case studies and long-term follow-up are needed to validate this observation.

## Conclusion

This case suggests that, similar to CA stenosis, stenosis or occlusion of the SMA may also alter blood flow in the pancreaticoduodenal arcade and contribute to aneurysm formation. The risk of rupture in SMA-associated aneurysms may be comparable to that of CA-associated aneurysms, and appropriate therapeutic interventions are warranted. The necessity of postoperative revascularization remains a subject for future investigation.
